# Exploring the Postpartum Experiences of Tamil Mothers Living in Canada: A Qualitative Study

**DOI:** 10.7759/cureus.107241

**Published:** 2026-04-17

**Authors:** Danica Kannathasan, Elena Neiterman

**Affiliations:** 1 School of Public Health Sciences, University of Waterloo, Waterloo, CAN

**Keywords:** maternal, maternal healthcare, postpartum, pregnancy, qualitative, south asian, tamil, women and health

## Abstract

Introduction

While the challenges experienced by immigrant and non-immigrant mothers during the postpartum period have been explored in the literature, there is a paucity of research on the postpartum experiences of Tamil mothers in Canada, whose transition to motherhood might be uniquely shaped by intergenerational trauma, cultural norms, and/or racial discrimination. This paper addresses this gap by examining Tamil mothers’ experiences during the postpartum period and identifies the challenges they face.

Research question

This research explores how Canadian Tamil mothers navigate motherhood during the postpartum period. Specifically, it examines (1) what support Tamil mothers need to help them navigate the postpartum period, and (2) what impact does the transition to motherhood have on Tamil mothers’ mental health and well-being?

Material and methods

A qualitative methodological design was employed to conduct the study. Using narrative inquiry, 15 semi-structured interviews were conducted with Tamil mothers across Canada to gain an in-depth understanding of their postpartum experiences. The interviews, lasting approximately 45 minutes, were conducted in English and Tamil. They were recorded and transcribed verbatim. Inductive reflexive thematic analysis was utilized to make sense of the data.

Results

The participants resided in Ontario (n=13) and British Columbia (BC; n=2), had one to three children, and most (n=11) were born outside of Canada. The findings from the study revealed that Tamil mothers have unique challenges during the postpartum period. Mothers living in Canada without family support require greater access to culturally sensitive community services. While those with family in Canada found it beneficial to rely on their (mostly) female relatives for practical and instrumental support, they also felt pressured to adhere to traditional cultural practices, which at times adversely affected their health. Participants who sought mental health and postpartum services believed that these services needed to be culturally sensitive to the unique needs of South Asian women in general, and Tamil mothers in particular.

Conclusion

Tamil mothers face unique challenges during the postpartum period, which can negatively affect their mental health and well-being. Culturally sensitive mental health and community outreach services, which target not only mothers themselves but also their extended family (mothers and mothers-in-law), might help Tamil mothers better navigate their postpartum transition.

## Introduction

The postpartum period, sometimes referred to as the “fourth stage of labor" [1,2], is an important and challenging time for everyone involved, especially mothers, as they recover from birth and adapt to their new role [3]. Primigravid mothers can find this period particularly difficult, especially when they lack family support and/or are uncertain about their ability to care for the newborn [4,5]. 

While all mothers may experience challenges during the postpartum period, immigrant and refugee women might be especially vulnerable during this life stage [4]. In addition to language barriers and knowledge about available community supports, refugee and immigrant mothers may also be separated from their extended family upon moving to a new country, which may significantly reduce their reliance on extended family support [4]. The postpartum period can also trigger the onset of mental health problems, with postpartum depression (PPD) affecting approximately 17% of mothers globally [6,7]. 

The need for the provision of support services for PPD and postpartum mental health has been identified and recognized as a health priority in many high-income countries [8]. Despite this recognition, there is a poor understanding of immigrant women’s psychological and social needs, which can make them more susceptible to PPD [8] and create additional difficulties for these mothers [8]. 

South Asian mothers, regardless of their immigrant status, have a higher risk of developing PPD [9,10]. The stigma attached to maternal mental health is detrimental, as family members and the community may not acknowledge the emotions that the new mother is experiencing [9]. South Asian mothers are expected to quickly adjust to the birth of a child and are discouraged from seeking mental health support [9]. Many South Asian women, including those living in high-income countries, may continue following traditional cultural practices during the postpartum period [11]. For this reason, family and community play prominent roles in providing support for mothers during the postpartum period; however, the intergenerational differences and cultural tensions can negatively impact new mothers’ mental health [12]. 

While research on South Asian mothers’ postpartum experiences is growing, for the most part, it tends to homogenize South Asians into one large group without recognizing the diversity of the South Asian community, which consists of many ethnic and religious groups with different cultural and traditional practices [9]. This paper focused on Tamil mothers, a distinct cultural and ethnic group of the South Asian diaspora living in Canada, to understand their experiences during the postpartum period. 

Canada is home to one of the largest Tamil diasporas [13], the oldest living ethnic group from South Asia, primarily from the northeastern parts of Sri Lanka and the southern parts of India [13]. According to the 2021 census, approximately 77,845 Tamil women were living in Canada [14]. Like most ethnic groups, Tamil mothers have cultural and traditional practices that can alter their postpartum period. For example, after giving birth, Tamil mothers are expected to follow the confinement period of 31-41 days [15]. During this time, they are taken care of by their families and follow a postpartum diet, which helps restore their health and increase milk production [15]. The postpartum diet consists of iron and calcium-rich foods [15]. For example, *Pathiya kulambu* is a popular herbal curry consumed during this period; this dish consists of a spice mix of cumin, fennel, turmeric, and black peppercorns, which are used to cook protein and vegetarian dishes [15]. Despite the unique expectations placed on Tamil mothers upon birth and the relatively large Tamil diaspora within Canada, limited research has been conducted on the postpartum experiences of Tamil mothers. Yet, their experiences may be unique due to many factors, such as the genocide in Sri Lanka and intergenerational trauma. The goal of this paper is to address this gap by exploring (1) how Tamil mothers living in Canada navigate the postpartum period and (2) how healthcare and community workers can support them. Given the growing diaspora of Tamil mothers in the global North, the findings from this study may contribute to the provision of culturally sensitive care. 

## Materials and methods

A narrative inquiry qualitative study design using semi-structured interviews was employed to explore Tamil mothers’ lived experiences. Participants were recruited in January 2023 upon receiving clearance from the Office of Research Ethics, University of Waterloo, Waterloo, Canada (approval number: ORE #44752). In total, 15 participants were recruited by circulating posters about the study on the Instagram accounts of two Canadian Tamil content creators. The posters sought participants who were Tamil mothers who had given birth in the last three years and were living in Canada.

Participants who contacted the researcher after seeing the posters and provided written consent to take part in the study were interviewed either by phone or via video call on Microsoft Teams (Microsoft Corp., Redmond, WA, USA). Although COVID-19 restrictions were lifted in Canada in 2023, during the time of the data collection, most participants expressed a preference for a virtual interview, and some were interviewed virtually due to geographic and logistical reasons. 

At the time of the interviews, participants were six to 24 months postpartum. The semi-structured interviews focused on participants’ experiences of transitioning to motherhood, the challenges they encountered, the formal and informal supports that helped them cope with postpartum challenges, and their recommendations for how healthcare and community workers could better support mothers during this vulnerable period (see Appendix A for the interview guide). Participant recruitment concluded once no new themes relevant to the study objectives emerged from the data, and thematic saturation had been reached.

The interviews were conducted in English or a mix of English and Tamil, and ranged from 15 minutes to 1 hour and 49 minutes, with an average interview time of 45 minutes. At the end of each interview, participants were asked to provide background information, including their age, number of children, immigration status, household size, highest level of education, and employment status. These demographic details were collected to contextualize participants’ narratives and enhance understanding of their experiences.

Data analysis

Interview transcripts were transcribed verbatim and underwent a series of preparatory steps, including cleaning, anonymization, and uploading to NVivo 12 (Lumivero, Denver, CO, USA) for analysis. When participants or the interviewer spoke in Tamil, the transcript was not transcribed verbatim; instead, it was translated for meaning. Reflexive thematic analysis, as outlined by Braun and Clarke, was utilized to generate themes from the data and organize the findings [16]. Their six-phase approach guided the analysis: (1) familiarization with the transcripts, (2) initial code development, (3) identification of themes, (4) review of potential themes, (5) defining and naming the themes, and (6) writing up the findings. Codes were developed inductively, allowing participants' experiences to shape the themes that emerged from the data. 

The reflexive thematic analysis integrated the lens of two researchers with different cultural and social backgrounds. The first author, who is a researcher with personal and cultural ties to the Tamil community, was reflexively relying on her shared linguistic, cultural, and gendered background while conducting interviews with participants. During the analysis process, the first author was able to co-construct knowledge that was both contextually grounded and ethically produced [16]. The second author of the study, a White, immigrant mother who is not from South Asia but who has expertise in research on pregnancy and motherhood, reflected on how her interpretation of the interview data is shaped by her personal background. During the analysis, both authors independently analyzed a subset of five interviews and discussed the generated themes, ultimately reaching consensus. 

## Results

The 15 Tamil mothers interviewed for this study were between the ages of 29 and 39 years old, and all were married. Six participants were first-time mothers, and nine had two or more children. Most participants (n=13) resided in Ontario, but two were from British Columbia (BC). The majority of the participants (n=11) were born outside of Canada. Participants’ education includes a college diploma (n=2), an undergraduate (n=6), or a graduate (n=7) degree. Most participants (n=11) lived in a nuclear household, while others (n=4) lived with their parents and/or in-laws. The participants’ demographics are summarized in Table 1.

**Table 1:** Participant demographics

Participant Number	Age (years)	Number of Children	Immigration Status	Marital Status	Number of People Living in the Household	Highest Level of Education	Employment Status	Location
1	32	1	Canadian	Married	3	Undergrad	Full time	Scarborough, Ontario
2	32	2	Canadian	Married	4	Masters	Full time	Langley, British Columbia
3	37	3	Canadian	Married	5	Undergrad	Full time	Mississauga, Ontario
4	31	1	Canadian	Married	3	Masters	Full time	Mississauga, Ontario
5	34	1	Canadian	Married	3	Masters	Full time	Burnaby, British Columbia
6	39	2 + 1 miscarriage	Canadian	Married	6	Undergrad	Full time	North York, Ontario
7	35	3	Canadian	Married	8	Diploma	Full time	Oshawa, Ontario
8	35	2	Canadian	Married	4	Undergrad	Full time	Pickering, Ontario
9	34	1	Canadian	Married	3	College	Full time	Pickering, Ontario
10	29	1	Canadian	Married	3	Masters	Full time	Ajax, Ontario
11	36	3	Canadian	Married	7	Undergrad	Full time	Kitchener, Ontario
12	34	3 pregnancy losses	Canadian	Married	2	MD General Surgery Resident	Full time	Toronto, Ontario
13	32	1	Canadian	Married	3	Masters	Full time	Toronto, Ontario
14	32	1	Canadian	Married	3	Masters	Full time	Mississauga, Ontario
15	34	2	Canadian	Married	4	Undergrad	Full time	East Gwillimbury, Ontario

**Figure 1:**
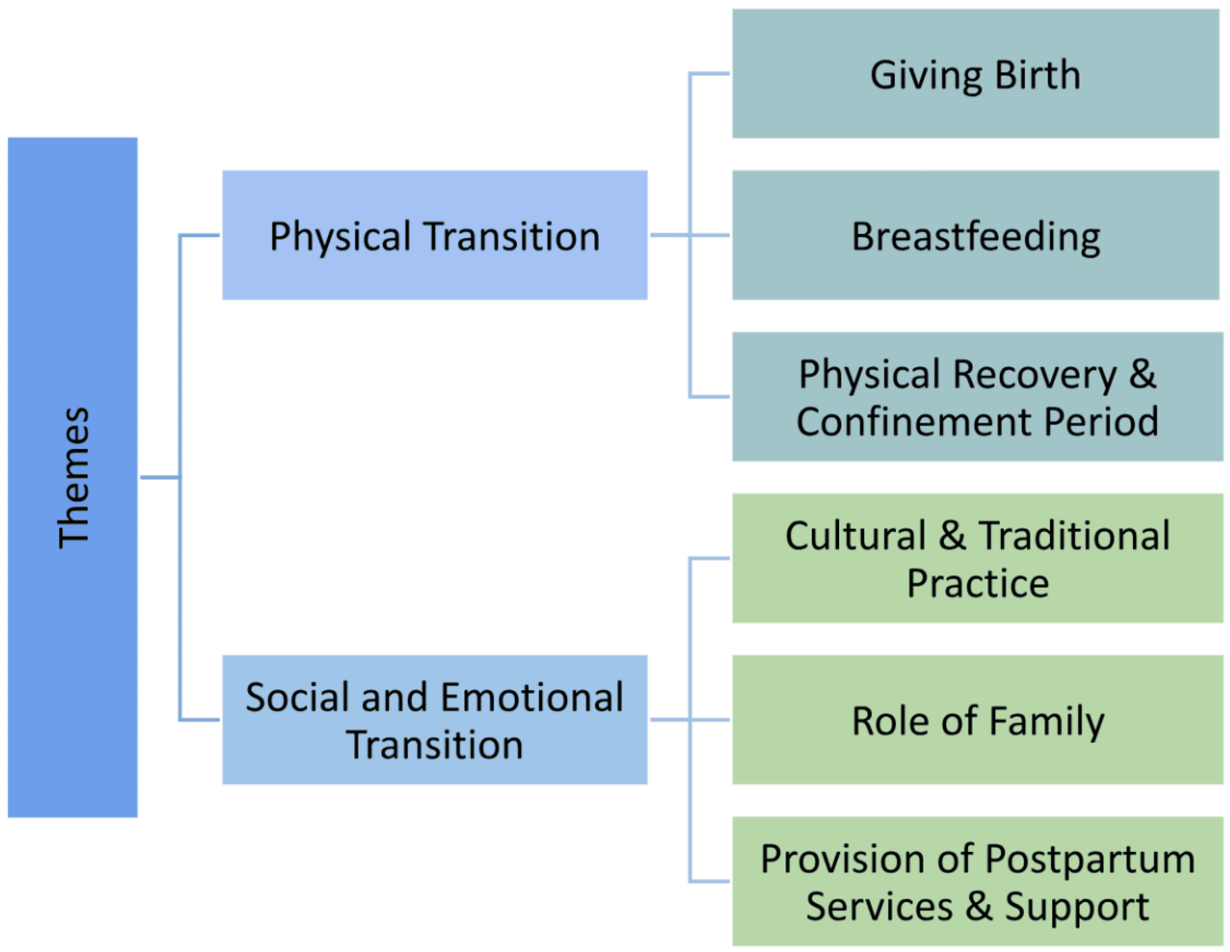
The overarching themes are physical transition and social and emotional transition.

The Tamil mothers in this study revealed that they experienced unique challenges during their postpartum period, which were broadly summarized into (1) changes associated with the physical transition of the postpartum period and (2) social and emotional transition. These two transitions served as overarching themes and were used to generate sub-themes summarized in Figure 1.

Physical transition

Sub-theme 1: Giving Birth

Since all participants (n=15) gave birth during the COVID-19 pandemic, their experiences of birth and the postpartum period were shaped by the lockdowns and restrictions placed on maternity wards in Canada during the time they were giving birth. Participants' accounts included stories about the challenges related to their inability to have their parents and children visit them while they were in the hospital, difficulty adjusting to the hospital policies that were frequently changing, trouble receiving the COVID-19 vaccine, and having to wear a mask during delivery. 

Participants recalled varied levels of support from hospital staff. Some participants said they felt supported, while others felt dismissed. Reflecting on their experiences, some participants attributed the negative attitudes of hospital staff to racism, while others saw them as a consequence of poor medical care or a combination of both of these factors. For example, Participant 12, a healthcare professional who had previous pregnancy losses, had a complication during the delivery and was not provided with proper care, which she believed to be a cause of her child’s death. Reflecting on her experience, Participant 12 exclaimed:

"If they did this to…a fellow colleague… if they treated me like that, what is another woman who's just coming in, assuming that the doctors are going to do the best for her and another woman of colour who's not as like, you know, empowered or fluent in, like, medical lingo or whatever, how is she going to be treated? You know what I mean? Like, I just found that so upsetting. Do you have to have a medical degree or a family member who's in medicine in order to get adequate care as a person of colour? (...) If my husband was White and he was standing there the whole time that they were going through all these things, I guarantee you the treatment would have been different, right? But my husband is a soft-spoken Brown man." 

In summary, while the participants noted that COVID-19 presented distinct challenges for receiving good care during delivery, they felt that their race/ethnicity introduced additional adversities, negatively shaping their birthing experiences.

Sub-theme 2: Breastfeeding 

Most participants believed that since breastfeeding is portrayed as an effortless process that comes naturally, it would not be a problem to breastfeed. However, many participants experienced challenges with breastfeeding, and two of the mothers were unable to breastfeed their babies, despite trying very hard. Recalling these challenges, Participant 8, a 35-year-old mother of two children, noted: 

"The whole breastfeeding experience was traumatizing for me. I think that media and, you know, all of that stuff (social media), like they glamorize it, and they make it seem like it is just a thing that needs to come naturally. You feel like you kind of failed as a mom. And there was nothing natural about it. I was crying. I was in pain." 

In addition to the media, mothers often had to deal with the pressures put on them by their family members. Participants noted that their inability to breastfeed was often seen by their family members as rooted in selfish and personal reasons, such as prioritizing their own beauty or convenience. Participant 13 shared: 

"It's like it's you're (..) you're considered to be like a lazy mom, for not breastfeeding. There's a huge like (..) it's a huge gap in our community, because like, we don't talk about that enough. And like, we don't talk about, like the mental impact that breastfeeding has on you as a mom, right? Like, when you feel like you're not producing enough milk because like that was (..) that was my case."

While most participants struggled with breastfeeding, two mothers described it as a non-stressful time. These mothers attributed their success to establishing boundaries with their extended family members early on. For example, Participant 7, a 35-year-old mother of three children, noted: 

"My family really didn’t pressure me a lot (..) I don't let my in-laws into my circle as much, so that doesn't affect me. I was able to like (..) me, and my husband were able to make the decisions for ourselves. So societal pressures, I don't think there was (..) there was much except for the whole breastfeeding journey. I didn't breastfeed as much as I did for the 1st, and they were OK with it because they were twins. I guess they lowered the standards."

Overall, participants who shared their breastfeeding journey felt that it was challenging, primarily due to internal and external pressures from society and their families. However, those who established boundaries with their family members found breastfeeding to be less stressful. 

Sub-theme 3: Physical Recovery and Confinement Period 

For many participants, physical recovery after giving birth was a challenging journey. Regardless of the type of birth the participants had, they shared that they did not have enough time to focus on their physical recovery. Many participants, particularly primigravid mothers, voiced feeling unprepared and unaware of the physical changes they would experience, since, as Participant 1 stated, “talking about the birthing process is not normalized” in the Tamil community. The following excerpt from Participant 1, a 32-year-old mother of a 4 ½ month old baby, is illustrative of the physical challenges faced by the participants: 

"How I pictured it in my head was that, okay, like, after the birth, I'm going to come home, and then me and my husband are going to, like tag team this, we are going to do this together. However, I didn't really (…) I didn't really realize how much recovery like I would have to go through before I could feel normal, like physically. So, I wasn't able to do everything. Like (…) till this day, I tell people like I felt like a part-time caregiver to my child because I only took care of my child for like, a few hours. Because it was either my husband or my mom or my mother-in-law, my sister-in-law, like people, other people, kind of stepping in and I felt like (…) that created like a lot of like, disconnect, I didn't feel like that bond that was supposed to be there formed. It was just like okay, what am I doing?" 

While some participants struggled with recovering physically from the process of birth and getting back their strength, others talked about the challenges of dealing with pelvic health complications. This topic was often silenced within the community. Two participants shared that they accessed pelvic physiotherapy and found it helpful as they were able to solve their concerns, such as incontinence. Participants did mention that they had to do their own research to find these services, as their OBGYN did not encourage or introduce pelvic physiotherapy to them. 

Tamil mothers are expected to follow the confinement period, a traditional Tamil cultural practice for postpartum mothers, which involves staying at home for 31-41 days and following a strict postpartum diet while being taken care of by female relatives [15]. Participant 12 shared: 

"For better or worse, the community is very good at… taking care of postpartum women in that immediate period… There's a lot of physical support, at least for postpartum women. Like I was saying, with nutritional support and the traditional foods that they give you around that time and (…) those 31 days of confinement is like this very protected time, right? Like, my mother, I remember her telling me that when she was in the 31-day period, it was just like, you just eat, you take care of your baby, and you breastfeed. So, like it's in some ways it's very protective. So, I think the community does a very good job of doing that and creating those supports within ourselves." 

The majority (n=10) of the participants followed this cultural practice, and almost all (n=14) followed the cultural practices surrounding the postpartum diet. In general, participants who followed the confinement period either loved or hated it. For instance, Participant 4, a 34-year-old mother of a 10-month-old infant, remarked:

"And I think having 31 days is almost like a blessing. Because, again, with COVID, not having to navigate life, and things like that, but also just having the time to do things at my own pace, wear clothes the way I want to wear it, you know, all those sorts of things. I don't have to dress up. I found that so helpful. And a great excuse for why I didn’t want guest right away, you know, I'd welcome people to see my child, but like, a little bit later on. The only thing I didn't enjoy as much was not being able to walk outside (..) I was just craving fresh air (..) I had to seek permission to do that. It wasn't like the make or break of the whole thing. Like, I actually enjoyed the 31 days of just keeping to myself and my family." 

Overall, the lengthy process of physical recovery came as a surprise for most participants in this study. Participants who followed the confinement period had both positive and negative experiences. Notably, while the confinement period aided in physical recovery, it fell short in providing emotional and mental support for the mothers, and some participants doubted they would practice confinement in their subsequent births.

Social and emotional transition

Sub-theme 4: Cultural and Traditional Practices 

Participants found that some cultural and traditional practices failed to consider their emotions. For instance, participants highlighted that in Tamil culture, when the baby is delivered, a flow of family members, including extended family, rush to the hospital to visit the newborn and mother. Many participants mentioned this was a tiring experience. 

During the COVID-19 pandemic, hospitals in Canada had strict regulations limiting the number of visitors. For the most part, participants were appreciative of the hospitals’ visiting policies, seeing it as a silver lining of COVID-19 restrictions. For example, Participant 15, a 34-year-old mother of two children (four years old and two years old), mentioned: 

"It is nice in the sense that there were no visitors, so I was able to actually rest as much as humanly possible and without like the constant stream of people. And you know, like Tamil people, they have to come to the hospital and (..) and we all have massive families. So, it's not just like three or four people. It's like 30-40, 50 people. The whole village."

Additionally, when family members visited the baby, participants found that they often made remarks about the baby’s appearance. The majority of participants (n=13) struggled with their family members, advising them to engage in cultural rituals and practices related to the newborn, such as head moulding. For example, Participant 13 mentioned: 

"Yeah, it's like the pillows, giving water to, you know, for a baby that's under six months. And like they used to press on the nose, and they press on the shape of the head. I was like “no, don't do that.” Oh, my God. It's kind of like fighting with them and telling them, like, don't do those things. Like, first of all, it's not even (..) like physically possible for you to like (..) maneuver the shape of a child's nose or a head. Like, what is this, like, play dough? It's a human being, you know what I mean?" 

The participants shared struggles they experienced trying to balance their cultural practices and religious traditions with medical and cultural norms they adopted in Canada. Some participants had established firm boundaries with their extended family members and found that they had more freedom in which cultural traditions they followed, but not all had a chance to exercise their own agency. 

Sub-theme 5: Family Support

Almost all (with the exception of one) participants received familial support during the postpartum period. The participants who had family members living in Canada highlighted that their families provided them with instrumental and psychological support. For instance, Participant 4 said: 

"I definitely was very lucky. Like the whole, ‘it takes a village to raise a child’, I had such a strong village on my side. So that included my mom, it included my siblings, my dad, my partner, and the friendship group that I have, even if it's not an experience that they have gone through, they were able to just empathize and be available." 

Despite receiving instrumental support, many participants felt that they did not have emotional support and that their family members or relatives simply assumed they were OK. 

While participants talked about how their family members could have been more emotionally supportive, Participant 15 shared that since she previously had postpartum depression with her firstborn, she valued and prioritized receiving mental support from her inner circle as opposed to receiving instrumental support: 

"They're (husband, parents, in-laws, siblings, grandma prior to her death, aunt, cousin) all very supportive when I need help. They are there immediately. (…) And then there are some friends. It's hard in the sense that they're not physically all able to help me, but the mental support, the mental aspect is what really is hard for me versus doing the things like changing diapers, waking up in the middle of the night. That stuff is not so challenging. (…) So having the ability to talk out my feelings with my support network is incredibly important. So, I'm very lucky and I know that."

Due to the COVID-19 pandemic and travel restrictions that were placed, Participant 5, who did not have family living in Canada, was unable to visit her family in India. While she felt sad about not having her family around, she also said that “it actually was helpful, in that I didn't have too many conflicting points of view (with other family members)”.  

The majority of participants (n=14) felt that they received a great deal of support from their spouses. For instance, Participant 10 recollected:

"From the moment we found we were pregnant, he's been there for me always. (...) And then, you know, through labour everything even afterwards. So, he took two months of parental leave, so he didn't work, and he was there. And even though I was going through my own like postpartum blues and everything. In those couple of first few months, like I really appreciated his help. (…) He would stay awake with me while I was breastfeeding her in the middle of the night, even though he didn't have to, because he felt bad, right? So those little things, they really meant so much to me. Even though I was going through my own kind of stuff, like, it was just nice to have him there and be there for me. Fast forward now, like after the initial phase of like having a newborn, he's just been, you know, so dependent on like, I can actually now go out with my friends and like, he'll take care of her, put her to sleep."

Although most participants relied on their families for instrumental and psychological support, some also felt strain in their relationships with family members as they negotiated care for the newborn, as is evident in the account of Participant 10: 

"They're (parents) like, ‘no, but this is what we did back home, and it worked’. And like, I get it, right. Like, that was the hard part because it's like I don't want to dismiss what they did and what happened to them because it worked for them. But things have changed. It was challenging because our daughter is the first baby for both sides of the family, so everything we did was new to them. My parents and my husband's parents were like, ‘what are you guys doing? This is weird. Like you don't have any toys in the crib?’ Like no, she's not supposed to have toys." 

Expanding on the challenges faced in family relationships during the postpartum period, Participant 14 noted:

"Tamil people come here (to Canada) because they were fleeing a war. Like, our parents were literally just surviving. They kind of know English. Some of them know English, some of them don't. But their only priority was like, okay, let's just make money and let's just get our kids educated. Like, it's really hard to tell your parents what to do when they raised five kids, and the kids turned out half decent. I find that with me and my husband, we both decided that we are going to respect our parents and no matter what they do, we will not say anything back. (…) I feel like every Tamil mom is doing it very different with their parents. And I don't know what the right answer is but I don't think we're doing the best job either." 

As a result of trying to teach their family members novel ways of raising an infant, participants felt that it was challenging to unlearn their cultural practices and adopt new ones. This difficulty arose as their mothers and mothers-in-law, who often share advice, tended to dismiss their suggestions. Consequently, they felt inferior when trying to figure out how to take care of their babies. For example, Participant 11 recounted: 

"I'm so scared that I may do something wrong. ‘I'm not getting enough milk to feed her. I'm not doing it right. Why is she crying? I don't know’. Like, I felt like I didn't know. And as much research you do, you still feel overwhelmed; when the babies crying and you're not able as a parent, especially as a mom, you're not able to comfort them. And so, my mother-in-law would be like...and even my mom too. They're like ‘உனக்கு தெரியாது’ (‘you don't know what you're doing’). Give the baby to me I’ll take care of him. Like automatically, they took the baby away from me (laughed), (..) instead of like helping me deal with it. And I think that alone hurt me because in my head I was like, so I'm old enough and mature enough to have a child. But I'm not mature enough to care for it? I think that hurt me a lot. Like the fact that I felt like my comfort or you know, the bonding that I should have had with my baby was kind of like taken away because you know, as soon as she was done feeding or as soon I was done giving her a bottle and I burped her, for example, they would just take her away, whether it's to sit with them or what not, they're like, ‘oh, you don't know what you're doing’."

Strain in familial relationships was also present when it came to pregnancy loss. Three participants talked about pregnancy loss and highlighted the lack of recognition and support provided by family and the community. Participants mentioned that since female fertility and becoming a mother are high expectations for Tamil mothers, they felt pressured by their family members and society to give birth to a living baby. Participant 12 highlighted that there is a term, Malady, in the Tamil language that is used in a pejorative manner to describe a woman who is unable to conceive. 

It is evident that participants faced challenges dealing with conflicting advice from their mothers and/or mothers-in-law. While family acted as a source of support, they also acted as a source of disagreement and stress. In terms of pregnancy loss, mothers felt unsupported as they were often expected to heal by themselves and faced immense pressure to be a mother to a living child. 

Sub-theme 6: Provision of Postpartum Services and Support

While some participants received postpartum support, the perceived effectiveness of these services varied among participants. Participants felt more supported when healthcare professionals were receptive to cultural traditions and provided advice on how to integrate cultural practices into postpartum care. Additionally, since Tamil culture places a lot of focus on food during the postpartum period, participants felt it was important to have their healthcare professionals give them more information about what kinds of food they should eat. However, participants found that in the Canadian healthcare system, there were no particular approaches to diet during the postpartum period, and healthcare professionals offered little advice on breastfeeding diet. 

Participants also expressed a desire to have educational resources on postpartum recovery, including strategies for offering emotional care, that could be shared with their extended families. For example, Participant 11 shared: 

"My mom or my mother-in-law, either one of them, would be more understanding and more willing to listen to someone else, that's closer to their age or older or someone that's in an authoritative position and told them, “hey, you know, these things happen. A mom does go through these stages in the first couple of weeks since birth. It's normal. We should be helping them, and you know (...) this is what we need to do. We need to respect the postpartum mothers’ opinions and if they're struggling, just be there, comfort them. Don't critique them. Don't tell them, ‘Oh you did this wrong’”. If they got that aspect, I think they’ll be more understanding and more willing to listen to them as opposed to me because the first thing that they do when the new mom says something, they're like, “oh, you just became a mom and already you know everything.”"

While participants found the resources related to caring for the baby helpful, most said they did not receive adequate information related to supporting their own mental health and well-being.

One of the participants who received pamphlets on postpartum care in the Tamil language found it helpful, as she was able to share these resources with family members. However, another participant said the translation was poor and thus did not see it as a helpful tool. 

Adding to the lack of culturally relevant care, participants mentioned that they were surprised by the lack of support they received from their OBGYN during their postpartum period. The mothers wished for more comprehensive care from their OBGYNs. 

The participants also believed that there was very little mental health support provided to mothers during the postpartum period. Participant 5 mentioned that “postpartum mental health support should be mandatory (…) because the likelihood you need it is higher than you don’t”. Echoing her, Participant 12 said: 

"I don't think the traditional healthcare system is set up to support women through the postpartum period, like the way that it's set up is they take care of you during pregnancy. You have one postpartum visit at six weeks...I think the traditional, like OBGYNs, is like the worst place to go to, like, seek out postpartum care. They're just not equipped for it. They don't care about it. That's not within their scope of practice."

However, in BC, Participant 2 mentioned that they received a call from a nurse at the 10-day mark to check in on them, and she found this very helpful:

"Here in BC, there's also like a follow up nurse call. I know, after COVID like it's on the phone. They call me but the first time when I had my first (child), they sometimes volunteer to come into your home to see how you're like breastfeeding or like, what issues you're facing (…) I would say like a preliminary not screening but it's more like preliminary, just checking to see if you're doing good if you know what you are doing (…) if you need additional like resources like for feeding and all that (…) that's one thing that I found really helpful." 

Seven participants mentioned that they saw lactation consultants, and three participants saw a therapist. Similar to OBGYNs, lactation consultants and therapists had mixed reviews; some participants found them helpful, while others did not. 

When accessing community support, the participants felt that they received the most help from community support professionals who were culturally competent. For instance, Participant 12 saw a psychiatrist after her pregnancy loss and remarked:

"I saw a psychiatrist (..) didn’t like (her), hated her. I felt like she gaslit me the whole time and her sort of objective was just to understand whether I need to be on, you know, antidepressants or not (..) umm, so that was a really, like, disempowering experience. And then I found a woman, brown woman therapist, who I found to be much better because a lot of the cultural things I didn't have to explain to her at all, it was just sort of understood."

In addition to in-person community support, participants also accessed virtual support. All the participants mentioned seeking support on social media, where they would connect with other mothers and/or healthcare professionals who discussed postpartum and baby health. 

Besides existing community supports, participants expressed the need for additional community-based interventions to support Tamil mothers during the postpartum period. They suggested offering home care and nutrition programs to address their specific needs. For example, Participant 12 remarked: 

"I think it would be helpful to have something that's more like you know, community centered, that's like culturally sensitive and actually based out of the needs of women of color and women, you know, Tamil women in particular...in terms of postpartum support, it needs to be community centered and not hospital centered and not healthcare centered for sure."

Overall, participants shared that culturally competent healthcare and community professionals were the most supportive. They suggested the need for additional community-based interventions that are designed to complement the care Tamil mothers receive from the Canadian healthcare system.

## Discussion

This study, which aimed to explore the experiences of Tamil mothers living in Canada during their postpartum period, showed that the transition to motherhood is uniquely shaped by Tamil cultural and ethnic identity. Participants mentioned that they relied on their family and/or spouses for instrumental and psychological support. Additionally, primigravid mothers who adhered to cultural and traditional practices found that their postpartum care was significantly influenced by their mothers and mothers-in-law. Many Tamil mothers also mentioned that they sought therapy, lactation consultations, pelvic therapy, and/or social media to help navigate their postpartum journey. It is important to acknowledge that the COVID‑19 lockdowns may have intensified some of these experiences; however, other challenges, such as discrimination or family‑related struggles, would have persisted regardless of the pandemic context.

In this study, most participants noted that the postpartum period impacted their mental health and well-being. Even though family provided support to the majority of the participants, family also caused strain. Entering motherhood as inexperienced mothers, participants often felt that they were pushed aside by their mothers and mother-in-law and had little agency when it came to taking care of their newborns. Participants also mentioned that they experienced a lot of pressure internally and externally to be the “perfect” mother. While pressure to be a “perfect” mother is commonly experienced by all mothers [17], the Tamil mothers in this study highlighted that being a mother is a big expectation in the Tamil community, as everyone is evaluating how well the mothers “perform”. In terms of internal pressure, the Tamil mothers mentioned that they felt an enormous responsibility to be the best mothers they could be. One of the main reasons for this internal pressure stemmed from the fact that most participants’ parents fled Sri Lanka due to the genocide. Despite the challenges they faced, these parents managed to navigate the process of acculturation while effectively raising their children. 

It is important to highlight that participants emphasized feeling more comfortable when interacting with healthcare or community professionals who demonstrated cultural competence and a willingness to listen. However, all 15 participants believed that in Canada, mothers’ mental health during the postpartum period is not prioritized. 

Additionally, the participants in the study highlighted various supports needed to help them during the postpartum period. These ranged from creating programs that provide healthy meals, since food plays a central role during this time, to having OBYGN appointments booked earlier than the conventional six-week mark. All the participants mentioned that the services and supports that were provided were tailored to the Western population; they wished that there were more culturally relevant community and health services available for Tamil mothers. They also suggested that their medical appointments should include primary caretakers who will be caring for the participant (usually mother or mother-in-law) during their postpartum recovery, as this will help reduce strain in their family relationships. In terms of pregnancy loss, a participant mentioned that there needs to be more community support available in the Tamil community. Currently, there is not a lot of support tailored toward mothers who have experienced loss, and they are expected to grieve alone and move on with their lives. 

A key finding from the study is that Tamil mothers’ ethnic background and gender intersect, which causes more adversities within the healthcare setting. Research has shown that implicit bias influences healthcare providers' actions toward Black, Indigenous, and People of Color (BIPOC) patients and creates health disparities based on factors such as gender and ethnicity [18]. This research supports this by showing that implicit bias is present among healthcare workers and negatively impacts Tamil mothers' experiences accessing healthcare services. 

Another key finding is that Tamil mothers maintain an uneasy balance between following traditional cultural practices and Westernized postpartum care. Many non-Western cultures are referred to as “ethnokinship” cultures, in which the postpartum support that is provided is social support-centric rituals that are performed for a longer duration than in Western cultures [19]. For Tamil mothers, the main postpartum tradition that they practice is the confinement period, which involves staying at home for 31-41 days, where the new mother is supposed to rest and is looked after by family members who generally are female (mother or mother-in-law) [15]. During this time, the new mother’s diet is restricted, and they are only allowed to eat a postpartum diet, which helps increase milk supply and heal the body [15]. The current literature highlights cultural variation in postpartum support; however, it fails to examine how most Tamil mothers in this study must negotiate which traditional practices they want to follow with their family members. It is important to highlight that participants agreed that families can be a very strong support system; however, they can add additional stress for Tamil mothers. Tamil traditional and cultural practices, such as giving water to the baby before they are six months old, may not be recommended by Health Canada and go against healthcare providers’ advice [20]. As a result, Tamil mothers experience strain in their familial relationships when they deviate from what their family members perceive as the norm.

The study has limitations. First, due to the small sample size, the findings are not generalizable to all Tamil mothers living in Canada. Another limitation is that we did not investigate how time spent in Canada could help or shift the Tamil mothers’ experience during postpartum. Future research can examine this by recruiting Tamil mothers who are recent immigrants to Canada vs. those who were born here to analyze how their postpartum experiences are similar and different.

Despite these limitations, this research provides valuable insights into the barriers Tamil mothers face during the postpartum period, which can help inform policymakers and healthcare professionals about their lived experiences. The findings also underscore the importance of training healthcare professionals, particularly those working in maternal health, on culturally sensitive care practices, including awareness of the role that family members may play in childbirth and postpartum recovery. Moreover, the study offers important recommendations for community and healthcare workers supporting Tamil mothers. These recommendations include: 1. Create culturally sensitive mental health and community outreach services, which target not only Tamil mothers themselves but also their extended families; 2. Train healthcare professionals to effectively communicate with Tamil mothers and advocate for their needs to their families; 3. Connect Tamil mothers to existing community programs that help support their mental health and well-being. For example, the City of Toronto has the Healthy Baby Healthy Children program that offers free home care services for mothers who meet its criteria [21].

Overall, Tamil mothers experience unique challenges during the postpartum period. To ensure they are supported, it is important for healthcare workers and community support to be culturally competent in the traditional and cultural practices that Tamil mothers follow. Furthermore, ethnically sensitive community services should be available for Tamil mothers.

## Conclusions

The postpartum period profoundly influences a mother's health, well-being, and social roles. For Tamil mothers, these experiences can be distinctly shaped by cultural beliefs, practices, and intergenerational trauma. It is imperative that healthcare and community workers possess cultural competence regarding postpartum traditions observed within the Tamil community. This competence is crucial for adequately supporting both primigravid and multigravida mothers as they navigate their postpartum journey. In addition to postpartum traditions, it's essential to implement support for mothers who experience pregnancy loss.

## Appendices

Appendix A

Interview Guide 

1. Tell me a little bit about the last time you gave birth. How long ago was it? What was the experience like?

2. How would you describe the time after you gave birth to your child?

          a. Probes: Was it how you expected? Did you feel prepared?

3. How has it been for you since the birth of your child?

          a. Probes: How has your life changed? How are you adjusting to this new life transition?

4. What factors do you think led to how you were feeling?

          a. Probes: societal pressure, expectations, timing of events.

5. Now that you have given birth to your child, who is supporting you in your role as mother?

          a. Probes: What is the role of your mother/mother-in-law? Can you describe your relationship with them?

          b. Probes: What role does your husband take on? What was your expectation?

          c. Probes: How do you deal with the different ways of receiving advice?

6. Could you describe how Tamil traditions, such as being homebound for 31-41 days (Thodukku), may affect the well-being of mothering during the postpartum period?

          a. Probes: benefits and implications

          b. Do you or anyone you know follow those traditions? In what way can they affect the postpartum experience?

7. Are you aware of any postpartum resources provided for the members of your community by health care providers?

          a. Have you ever accessed/received those resources? Were they helpful? In what way they were/not?

          b. (If accessed/familiar) Did you find them culturally relevant?

8. How do you think healthcare professionals or community leaders can support you during the postpartum period?

          a. Probes: mental health, well-being, physical health

9. Is there something that we did not talk about that you would like to add?

          a. (If not shared yet) To better understand the postpartum experience of Tamil mothers, can you please provide me with the  following information:

            · Age:

            · Number of children:

            · Race/ethnic background (mother):

            · Immigration status:

            · Marital status:

            · Number of People living in the household:

            · Highest level of education (mother):

            · Employment status (mother):
